# The Electrochemical Performances of n-Type Extended Lattice Spaced Si Negative Electrodes for Lithium-Ion Batteries

**DOI:** 10.3389/fchem.2019.00389

**Published:** 2019-05-31

**Authors:** Moonsang Lee, Dockyoung Yoon, Uk Jae Lee, Nurzhan Umirov, Aliya Mukanova, Zhumabay Bakenov, Sung-Soo Kim

**Affiliations:** ^1^Korea Basic Science Institute, Daejeon, South Korea; ^2^SK Innovation, Daejeon, South Korea; ^3^Graduate School of Energy Science and Technology, Chungnam National University, Daejeon, South Korea; ^4^School of Integrative Engineering, Chung-Ang University, Seoul, South Korea; ^5^National Laboratory Astana, School of Engineering, Nazarbayev University, Institute of Batteries, Astana, Kazakhstan

**Keywords:** lithium-ion batteries, silicon, dopant, arsenic, discharge capacity, retention

## Abstract

The electrochemical performances of lithium-ion batteries with different lattice-spacing Si negative electrodes were investigated. To achieve a homogeneous distribution of impurities in the Si anodes, single crystalline Si wafers with As-dopant were ball-milled to form irregular and agglomerated micro-flakes with an average size of ~10 μm. The structural analysis proved that the As-doped Si negative materials retain the increased lattice constant, thus, keep the existence of the residual tensile stress of around 1.7 GPa compared with undoped Si anode. Electrochemical characterization showed that the As-doped Si anodes have lower discharge capacity, but Coulombic efficiency and capacity retention were improved in contrast with those of the undoped one. This improvement of electrochemical characteristics was attributed to the increased potential barrier on the side of Si anodes, inherited from the electronic and mechanical nature of Si materials doped with As. We believe that this study will guide us the way to optimize the electrochemical performances of LIBs with Si-based anodes.

## Introduction

Lithium-ion batteries (LIBs) have been considered as an important energy storage device for modern portable electronics and electric vehicles (Goodenough and Park, 2013; Nitta et al., 2015; Kim et al., 2016). Until now, the graphite is typically used as anode material in commercial LIBs. However, its theoretical capacity (372 mAh g^−1^) is not sufficient for the next-generation mobile applications requiring high energy density (Peng et al., 2010; Ma et al., 2014; Ko et al., 2015). To overcome the energy density problem of current LIBs, the use of Si as the anode in LIBs can be an alternative way due to its unique physical properties such as high theoretical capacity of 3,580 mAh g^−1^ (Li_15_Si_4_ state) and low discharge potential (< 0.5 V vs. Li/Li^+^) (Wang and Dahn, 2006; Liu et al., 2012; Shi et al., 2016). Even if the Si material has such good advantages for LIBs, the commercial success of LIBs with Si anodes has been impeded by fast capacity fading of Si anodes. This fading results from large volume change more than 300% of Si anodes during lithiation and delithiation, leading to their disintegration (Ryu et al., 2004; Kim et al., 2011). Furthermore, semiconductor characteristics of Si make it even more difficult to be utilized in the practical application of LIBs as a negative electrode owing to the electrical losses due to large ohmic resistance (Wen and Tian, 2013; Wang et al., 2015).

Recently, various research groups have reported that the electrochemical performances of LIBs with Si anodes can be improved by using Si/Metal alloy electrode (Umirov et al., 2019a,b), the formation of nanostructured-anodes (Mukanova et al., 2018a,b), hollow and yolk-shell structured-composites (Yao et al., 2011; Wang et al., 2013), and doping of Si with impurities such as copper (Cu) (Wen and Tian, 2013), arsenic (As) (McSweeney et al., 2014), aluminum (Al) (Legrain and Manzhos, 2015), silver (Ag) (Talla et al., 2015), boron (B) (Long et al., 2011; Rousselot et al., 2012; Yi et al., 2013), nitrogen (N) (Han et al., 2016), and phosphorus (P) (Domi et al., 2016). Among other approaches, incorporating dopants into Si architectures has gained considerable attention as a suitable route for improving the rate capability, electrical conductivity, and capacity retention of the Si anodes (Mukanova et al., 2018b).

To date, many methods have been proposed for the Si powder preparation, including solution and solid-state based techniques, etc. Taking into account that the electronic/silicon-based semiconductor technologies have widely entered into society and industry, it is easy to imagine how much semiconductor class Si containing waste is produced every day. These silicon materials can be recycled for use in LIBs.

In this paper, we used the Si wafer in order to demonstrate the possibility of the Si electronic wastes to be reused in LIBs. As-modulated Si micro-flake anodes were investigated as an anode. To achieve a homogeneous distribution of As-impurity in the Si anodes, As-doped Si wafer was prepared by ball-milling and employed for the anode in a lithium cell. Moreover, this work covers the comparative analysis in the structural and electrochemical responses of As-doped and undoped Si microflake anodes to investigate the effects of the lattice spacing in Si anodes.

We believe that this paper will shed lights on the trends in the electrochemical performance [cycle retention, Coulombic efficiency (CE), and rate capability] of LIBs with the Si microflake anode with different lattice spacing.

## Experimental

The commercial As-doped single crystalline (100) Si wafers with a doping concentration of 1 × 10^19^ cm^−3^ were provided by SK Hynix and used as the anode material for LIBs. The doping concentrations of Si wafer were confirmed by secondary ion mass spectrometry (SIMS) (not shown in this paper). Furthermore, the intrinsic (undoped) Si wafers were used as the control samples to compare the effects of As doping. To remove native oxide on Si surface, Si wafers were immersed in diluted 10% hydrofluoric acid solution for 3 min and followed by rinsing in deionized (DI) water of ultra-purity (resistance 18.2 MΩcm) for 5 min. Si wafers were mechanically grounded to achieve Si microflakes using zirconia balls for 2 h in paint shaker (KM-2000T, Nara Sci).

The undoped and As-doped Si based electrodes were fabricated via direct casting of slurry onto Cu foil using the Dr. Blade method. The slurry was prepared by thoroughly mixing of active material and PAI (Polyamide-Imide, Solvay Torlon 4000T) binder with the wt.% ratio of 9:1, respectively, in NMP (N-Methyl-2-pyrrolidone, Merck) solvent. After casting the electrodes were dried in convection oven at 110°C to remove NMP. The loading level of electrodes were adjusted to 5 mg/cm^2^.

Electrochemical performances were studied using coin-type half configuration cells with Li metal as the counter electrodes, which were assembled in an Ar-filled glove box with < 1 ppm of both oxygen and moisture. The electrolyte solution used was 1.3 M lithium hexafluoro phosphate (LiPF_6_) dissolved in a solvent mixture of ethylene carbonate (EC) and diethyl carbonate (DEC) with a 3:7 volume ratio. Galvanostatic measurements were carried out to determine charge-discharge capacity and cycling retention in the potential range between 0.005 and 2.000 V vs. Li/Li^+^ under a current density of 1.0 A g^−1^ (0.28 C) using a computer-controlled battery measurement system (TOSCAT 3000 U).

The surface morphologies of Si microflake anodes were evaluated by field emission scanning electron microscope (FE-SEM). The stress evolution, phase transition, and crystallinity of Si microflake anodes were investigated by micro Raman spectroscopy and X-ray diffraction (XRD).

## Results and Discussion

**Figure 1:**
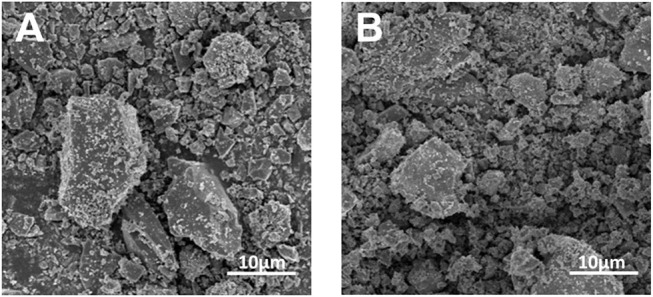
Plan-view SEM image of **(A)** undoped and **(B)** As-doped Si powders after ball-milling.

Figures 1A,B shows the plan-view SEM images of undoped and As-doped Si negative electrodes, respectively, after ball-milling for 2 h. The irregular and agglomerated particles were made up with the average size of 10 μm for both undoped and As-doped Si anodes. The particle size and distributions were measured by particle size analyzer (not shown in this paper). The similar morphology and size of micro particles of undoped and As-doped Si anodes were observed, indicating that As-dopants do not influence the morphology and particle size distribution of negative anodes.

**Figure 2:**
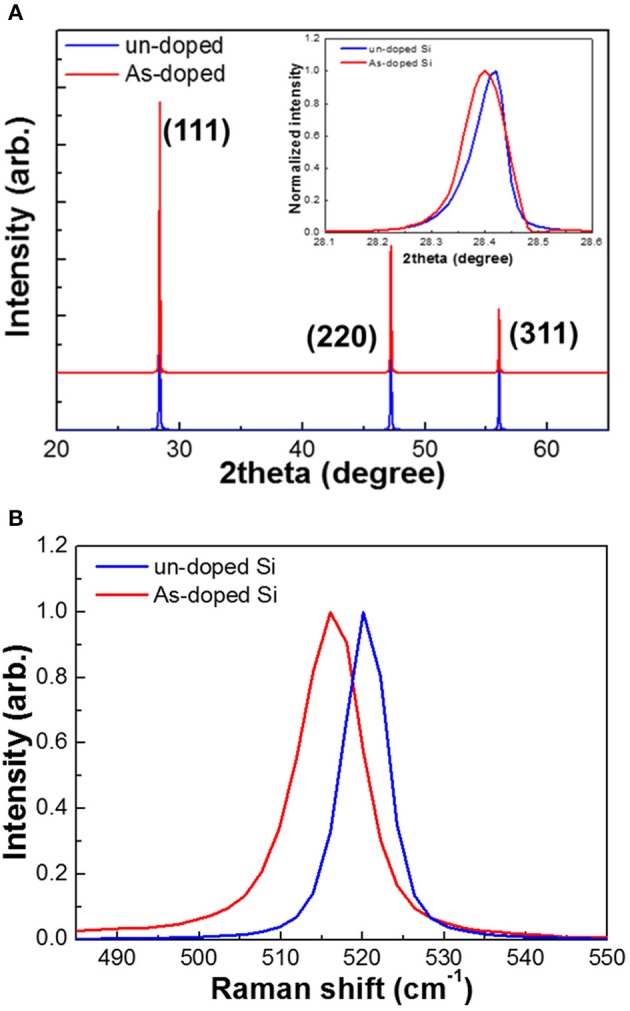
**(A)** XRD patterns and **(B)** Raman spectra of undoped and As-doped Si anodes.

Figure 2 exhibits XRD patterns and micro-Raman spectra of undoped and As-doped Si negative electrodes. The phase and crystallinity of Si anodes used in this work were investigated by XRD as shown in Figure 2A. It should be noted that all XRD peaks are sharp. This feature could be ascribed to the property of single crystalline Si. It is found that all the peak positions of XRD patterns can be indexed to those of cubic Si. It is interesting to note that the peak positions of As-doped Si anodes were shifted to the lower angles, which is attributed to the increased lattice constant by As-doping into Si structure as depicted in the inset of Figure 2A. Typical stress profiles of undoped and As-doped Si negative electrodes were studied by micro-Raman spectroscopy measurements at room temperature, as shown in Figure 2B. It is well-known that F_2g_-phonon modes in crystalline Si can be identified with peaks at around 520 cm^−1^ (Shimizu et al., 2015). We can easily notice that the peak position for the As-doped Si anode has been red-shifted, indicating the residual tensile stress of approximately ~1.7 GPa compared to the undoped one. The residual stress can be calculated using the following equation (Wu et al., 2007):

(1) $$\begin{array}{r} \sigma \left(\text{MPa}\right)=434\times \Delta \omega \end{array}$$

where σ is the biaxial stress, Δω is the F_2g_ phonon peak shift. These data are in good agreement with the XRD results in Figure 2A.

**Figure 3:**
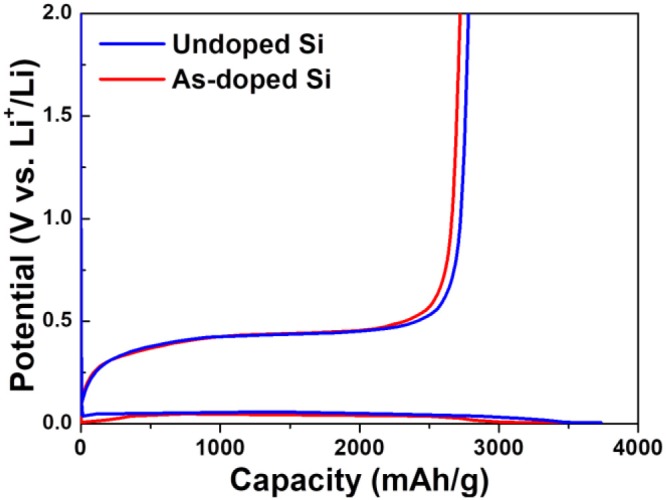
Typical galvanostatic first charge-discharge curves of undoped and As-doped Si electrodes in the potential range from 0.005 to 2.000 V vs. Li/Li^+^ at 1.0 A g^−1^.

In order to evaluate the electrochemical characteristics of Si negative electrodes with and without dopants, the various electrochemical examinations were performed. Figure 3 illustrates the first charge and discharge behavior of undoped and As-doped Si thick film anodes. It is clearly seen that there are plateaus in the curves, which is attributed to lithiation and delithiation by the reactions of Si atoms with Li ions (Chevrier et al., 2010). The initial charge potential plateau is located near 0.1 V, which is in a good agreement with the formation of Li_x_Si via lithiation of crystallized Si (Han et al., 2016). The first charge and discharge capacity for undoped and As-doped Si negative electrodes are 3,733, 2,782, 3,578, and 2,722 mAh g^−1^, respectively. These values correspond to the initial CE of 74.5 and 76.1%, respectively.

**Figure 4:**
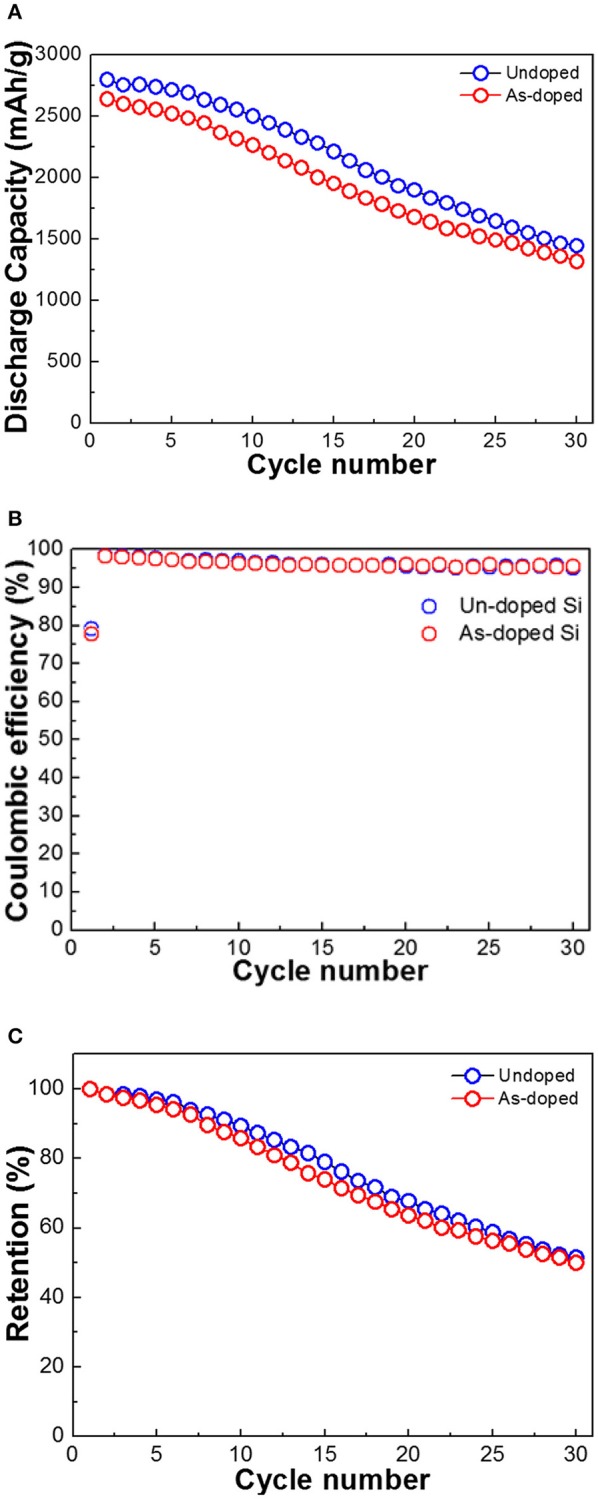
Electrochemical performance of undoped and As-doped Si anodes: **(A)** cycle performance, **(B)** Coulombic efficiency, and **(C)** capacity retention of negative electrodes vs. Li/Li^+^ at 1 A g^−1^.

Figure 4A illustrates the cycle performance of the cells with the undoped and As-doped Si negative electrodes. The discharge capacities of the LIBs with both types of the anodes were evaluated in a cut-off potential range of 0.005–2.000 V as seen in Figure 3. The discharge capacity of LIB with undoped Si negative electrode was higher than that of As-doped Si in all cycle ranges. One can easily observe that the discharge capacity fading of undoped Si anode is comparable with that of As-doped Si. Notably, a fading rate of discharge capacity with the undoped Si negative anode changed more sharply near 10 cycles. The initial discharge capacities of both anodes were decreased by approximately 50% after 30 cycles and their discharge capacities arrived at similar extent. Figure 4B shows the CE of both investigated materials. The subsequent cycle CE of cells with undoped and As-doped Si anodes start from 98.5 and 99.6% and further reaches over 95.0 and 95.8% after 30 cycles, respectively. In addition, the capacity retentions of both cells were measured as shown in Figure 4C. Despite the discharge capacity of undoped Si anode was higher than that with As-doped Si one, its capacity retention displays similar trends, corresponding to about 50% of retention. This phenomena can be explained by the fact that the As-doped Si anodes consumed more Li-ions for the generation of a solid-electrolyte interface (SEI) compared with the undoped Si anode and high energy barrier in energy band diagram, which will be mentioned later.

**Figure 5:**
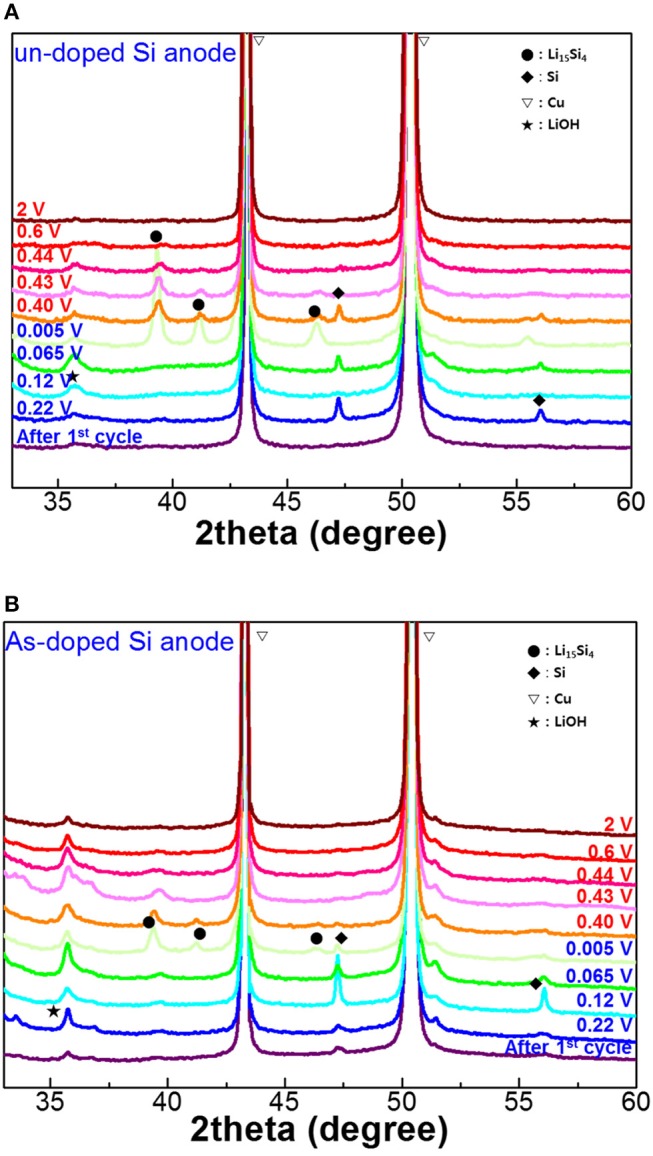
XRD patterns of **(A)** undoped and **(B)** As-doped Si anodes after the first lithiation/delithiation as a function of discharge/charge cut-off voltages. The red letters represent discharge voltages, and the blue ones charge voltages.

Figure 5 depicts 2theta X-ray rocking curves of the undoped and As-doped Si negative electrodes with various cut-off voltages (OCV) in charging/discharging states after the 1st cycle. After the formation process, there is no sign of the Li_15_Si_4_ phase presence in XRD curves for undoped and As-doped Si anodes. Besides, XRD patterns of both undoped and As-doped Si anodes discharged up to a potential of 0.005 V clearly revealed the presence of the peaks at 39° and 41°, indicating the formation of Li_15_Si_4_ silicate (Li and Dahn, 2007). It is essential to note that the intensity of XRD patterns of undoped Si negative electrodes was much higher than that of As-doped Si one. This implies that the phase transition of undoped Si anodes into Li_15_Si_4_ phase is easier to occur compared to the doped electrode. In the discharging states, the peak intensity of Li_15_Si_4_ reduced steadily with increasing the OCV, and finally disappeared at OCV of 2 V for undoped and As-doped Si anodes.

**Figure 6:**
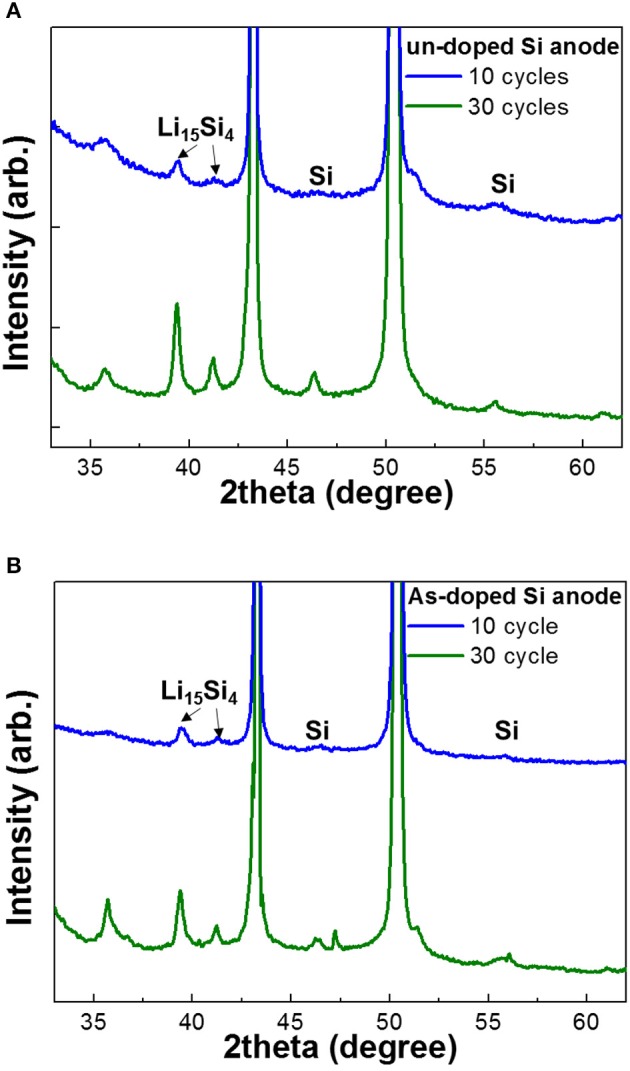
XRD patterns of **(A)** undoped and **(B)** As-doped Si anodes vs. cycles.

In order to estimate the phase transition of undoped and As-doped Si anodes, we performed XRD analysis as a function of cycle number as illustrated in Figure 6. No Li silicide peaks were found in the XRD spectra of the samples before cycling (not shown in this paper). The Li_15_Si_4_ phase peaks in XRD curves after 10 and 30 cycles were easily confirmed as seen in Figures 6A,B. Indeed, the XRD intensity of Li_15_Si_4_ phase in undoped Si anode is much higher than that in As-doped Si, which is consistent with the results obtained in Figure 5. In addition, the gradual increase of Li_15_Si_4_ phase upon 30 cycles is assigned to the activation process of Li-ion pathway in the electrode-electrolyte system, despite the fact that activation was accompanied by the fast capacity fading of both electrodes as observed in galvanostatic cycling.

**Figure 7:**
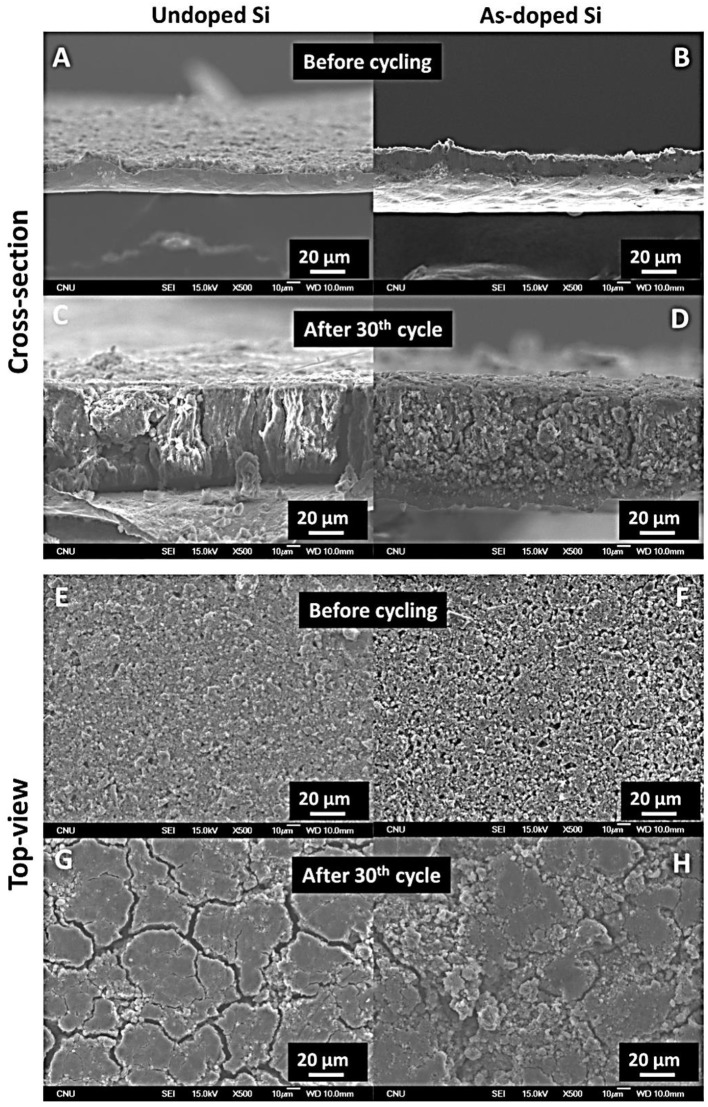
Cross-section SEM micrographs of undoped and As-doped Si anodes **(A,B)** before and **(C,D)** after 30th cycle. Top-view SEM micrographs of undoped and As-doped Si anodes **(E,F)** before and **(G,H)** after 30th cycle.

Furthermore, we investigated the morphology of the electrodes at their cross-section and surface before and after 30 cycles using SEM imaging. Cross-sectional SEM images of undoped and As-doped Si (Figures 7A,B) showed that both electrodes have the thickness of approximately 20 μm before cycling, while after 30 cycles, thickness increased drastically (>200%) that can be observed in Figures 7C,D. Besides, both samples exhibited similar changes in volume expansion. From the top-view images of undoped Si vs. As-doped Si (Figures 7E–H), one can see that the morphology of electrodes looked the same before and after cycling. The extensive formation of cracks and partial pulverization of active electrode material after 30 cycles was observed in both cases shown in Figures 7G,H. Accordingly, SEM results supported the capacity fading phenomena revealed by the galvanostatic tests earlier for the undoped and As-doped Si anodes.

**Figure 8:**
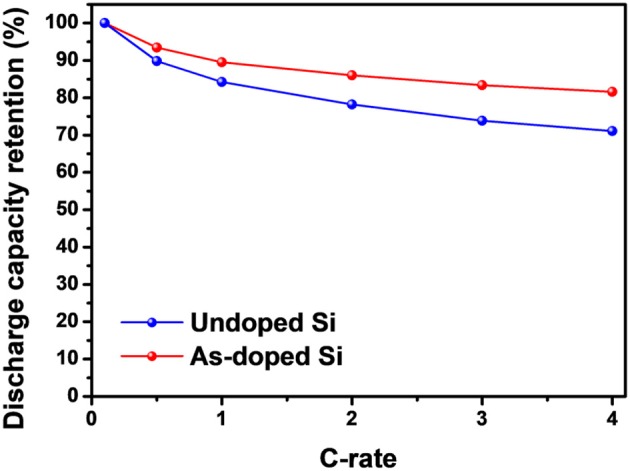
Rate capability of undoped and As-doped Si electrodes from 0.1 to 4.0C (1C = 1.0 A/g).

Figure 8 compares the discharge capacity retentions of undoped and As-doped Si electrodes at various current densities from 0.1 to 4.0 C (1C = 1.0 A/g), respectively. Here, undoped Si exhibits considerable fading in discharge capacity at higher current densities compare with As-doped Si. More specifically, at the highest current density of 4.0 C, the undoped Si-based electrode shows 69% in capacity retention vs. 0.1 C. By contrast, the As-doped Si demonstrates better rate performance with ~80% capacity retention. Obvious improvement in rate capability can be ascribed to the As-doping effect in Si structure and will be further discussed with the electronic energy band diagram.

**Figure 9:**
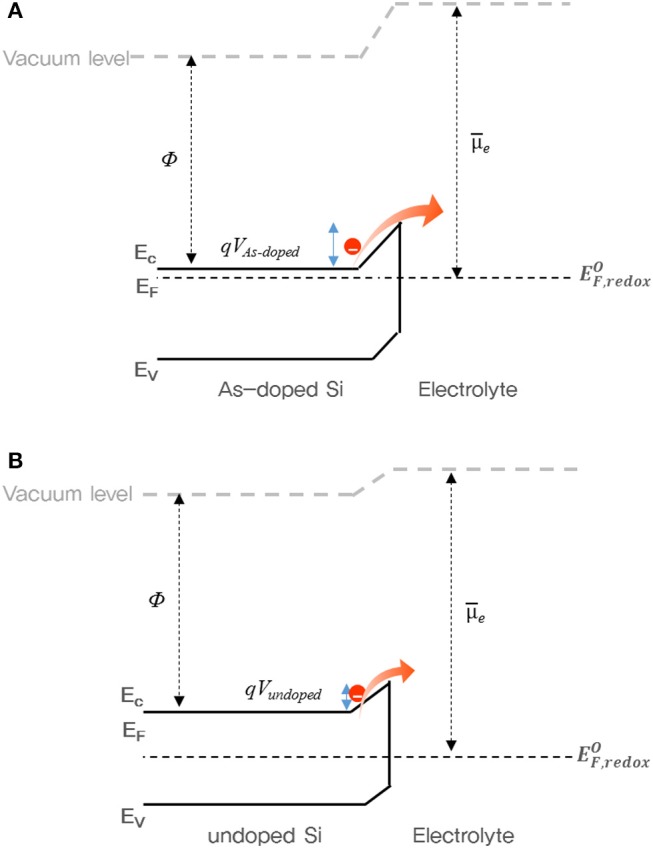
Energy band diagram of cells with **(A)** As- and **(B)** undoped Si negative electrodes during charging state. E_c_, E_v_, E_F_, E_F, redox_, and *U*_e_ indicate the conduction band edge, valence band edge, Fermi level, redox fermi level of electrolyte, and electrochemical potential of the electrolyte.

To understand the charge/discharge characteristics of undoped and As-doped Si negative electrodes, we took the electronic energy band diagram of both Si anode-electrolyte interfaces into consideration as illustrated in Figure 9. It is well-known that when the semiconductor-electrolyte interface is formed, electrolyte and semiconductor reach electrochemical equilibrium by matching their electrochemical potentials. This occurs by charge transfer between both media (Prados et al., 2014). Considering this, each barrier heights of undoped and As-doped Si anodes are represented in Figures 9A,B, respectively. It is noteworthy to mention that the energy barrier height from As-doped Si anode side, qV_As−doped_, was found to be higher than qV_undoped_ of undoped Si anode after equilibrium state. Hence, the flux of electrons across the electrolyte in As-doped Si with the increased potential barrier height can be impeded, thus hindering charge flow in the cell. In previously published works, the addition of dopants such as B and P decreased the lattice spacing of Si anodes and led to the increase of the insertion energy of Li ions, thus suppressing the discharging and charging capacity in LIBs (Long et al., 2011; Domi et al., 2016). However, the charging/discharging capacities of As-doped Si anodes have a different aspect, apart from volume change. To understand the electrochemical behaviors of cells with As-doped Si anodes, the energy band diagrams were illustrated, as shownv in Figure 9. It was found that the potential barrier of cells with As-dopant is much higher than that of undoped Si anode. We can infer that the higher potential barrier on the anode side by highly n-type doping results in the reduced charging/discharging capacity of As-doped Si anodes, compared to that of undoped Si ones. This is in a fair agreement with the results of Figures 5, 6. Moreover, we consider that slightly higher CE of As-doped Si negative electrode is related to the potential barrier. A lower charge/discharge capacity leads to the suppression of the phase transition from c-Si to a-Si, which may result in the improved CE, corresponding to the XRD results.

## Conclusion

In summary, the electrochemical performance of the cells with As-doped Si negative electrodes was investigated. The ball-milling of single-crystalline Si wafers was used to fabricate the anodes, which particles were irregular and agglomerated micro-flakes. The XRD and Raman analysis revealed that As-doped Si anodes have larger lattice spacing compared to undoped Si samples, indicating the residual tensile stress of approximately ~1.7 GPa. Despite the increased lattice spacing in As-doped Si anode, its charge/discharge capacity was lower compared with that of undoped Si anodes. The Coulombic efficiency and cyclic retention of As-doped Si negative electrodes, however, were comparable to those of the undoped Si anode. This effect was attributed to the increased potential barrier height on the side of As-doped Si, affecting the electrochemical performance of LIBs with the As-doped anode. We believe that this study will provide an effective approach to achieve the enhanced electrochemical performances of LIBs with Si-based anode derived from electronic waste materials.

## Data Availability

The datasets generated for this study are available on request to the corresponding author.

## Author Contributions

ML and DY conceived design of the study and carried out the experiment; UL, ZB, and S-SK contributed to the interpretation of the results; NU, AM, ZB, and S-SK wrote sections of the manuscript. All authors contributed to manuscript revision, read and approved the submitted version.

### Conflict of Interest Statement

The authors declare that the research was conducted in the absence of any commercial or financial relationships that could be construed as a potential conflict of interest.
